# Draft Genome Sequence of *Pseudomonas* sp. Strain JMM, a Sediment-Hosted Environmental Isolate

**DOI:** 10.1128/genomeA.00879-14

**Published:** 2014-09-04

**Authors:** Simmi Grewal, Jyoti Vakhlu, Vipin Gupta, Naseer Sangwan, Puneet Kohli, Namita Nayyar, Pooja Rani, Shivani Singh Sance, Rup Lal

**Affiliations:** a School of Biotechnology, University of Jammu, Jammu, India; b Department of Zoology, University of Delhi, New Delhi, India

## Abstract

*Pseudomonas* sp. strain JMM was isolated from the sediments of a natural water reservoir (pH, 6 to 7) located at Chambyal village in Samba district of Jammu and Kashmir, India. Here we report the annotated draft genome sequence of strain JMM having 52 contigs with 5,884 genes and an average G+C content of 66.5%.

## GENOME ANNOUNCEMENT

The genus *Pseudomonas* is a metabolically versatile and cosmopolitan taxon found in diverse ecological niches, including cold deserts, soil sediments, clinical samples, and rhizospheres (1–3).

In recent years, xenobiotic potential and metabolic versatility of *Pseudomonas* genotypes have gained a scientific focus that has resulted in the formation of a comprehensive *Pseudomonas* genome database (4). However, the relative contribution of freshwater isolates in this database is very low (4). To partly bridge this gap, we selected the strain JMM (a freshwater sediment isolate) for whole-genome sequencing.

Total genomic DNA of strain JMM was extracted and purified using QIAamp DNA MiniKit (Qiagen, Germany). The whole-genome sequence was generated using the Illumina HiSeq 2000 sequencing platform by constructing 2-kb and 500-bp paired-end libraries. Total sequencing data (4.80 Gbp, *n* = 23,613,000; 90 bp/read) were assembled into contigs (*n* = 52, >500 bp) by using the ABySS version 1.3.3 assembler (5) set at a k-mer size of 63, obtaining an *N*_50_ length of 389.7 kb with the largest contig being 668.9 kb. Contigs were evaluated for misassembly using a paired-end criterion, i.e., mapping raw reads using the sam-pe function implemented in Burrows-Wheeler Aligner (bwa) version 0.5.9. (6). Protein-encoding genes, tRNA genes, and rRNA genes were predicted using Glimmer version 3.02 (7), tRNA_scan-SE (8), and RNAmmer (9), respectively. A total of 5,884 coding DNA sequences (CDS), 71 t-RNA genes, and rRNA genes (5S, *n* = 4; 16S, *n* = 8; and 23S, *n* = 8) were observed with an average G+C content of 66.5%. Genome assembly was annotated using RAST version 4.0 (10), the NCBI Prokaryotic Genomes Annotation Pipeline (PGAP; http://www.ncbi.nlm.nih.gov/genomes/static/Pipeline.html), and the KEGG Automatic Annotation Server (KAAS) (11). The presence of 105 of the total 107 essential single-copy genes in the draft genome confirmed near-complete (98.13%) genome assembly (12). Pairwise whole-genome average nucleotide identity (13) analysis revealed that *Pseudomonas aeruginosa* strains, i.e., YL84 (99.50%) (14), PA38182 (99.44%) (accession no. HG530068.1), and SCV20265 (99.57%) (15) are the closest genetic neighbors of strain JMM. Twenty-one transposons (IS3, *n* = 19; and Tn3, *n* = 2) were predicted using BLASTp (E value = 10^−5^) (16) analysis against the Insertion Sequence (IS) Finder database (17). The PHAST online server (18) predicted three prophage regions of sizes 12.4 Kbp, 30.7 Kbp, and 17.7 Kbp having 8, 34, and 12 CDS, respectively. RAST-based (10) annotation revealed 117 protein-coding genes for the degradation of hydroxybenzoate (*n* = 9) and homogentisate (*n* = 14). The genetic presence of cobalt, zinc, and cadmium (*n* = 33) and multidrug-resistance proteins (*n* = 20) highlighted the heavy metal- and drug-resistance potential of strain JMM. The arsenic-resistance genes *arsR* and *arsA* and the ACR3 protein were also observed in the draft genome. A complete multidrug-resistance tripartite system and a BarA-UvrY (SirA) two-component regulatory system were also annotated in the genome. BLASTn (E value = 10^−5^) (16) results confirmed the presence of *rhl*- and *las-*based quorum-sensing systems, highlighting the population-dependent growth regulation potential of the strain. The analysis of the draft genome sequence of strain JMM coupled with already existing genomes of freshwater isolates of the genus *Pseudomonas* can now provide information to better understand environmental specific traits.

### Nucleotide sequence accession number.

The draft genome sequence of *Pseudomonas* sp. strain JMM has been deposited at GenBank under accession number JMQH00000000.
